# Air Pollution, Political Corruption, and Cardiovascular Disease in the Former Soviet Republics

**DOI:** 10.5334/aogh.3702

**Published:** 2022-07-01

**Authors:** Benjamin M. Varieur, Samantha Fisher, Philip J. Landrigan

**Affiliations:** 1 Global Public Health and the Common Good, Boston College, 140 Commonwealth Ave, Chestnut Hill, MA, US

**Keywords:** air pollution, fine particulate matter (PM_2.5_), Eastern Europe, Soviet Union, corruption, cardiovascular disease

## Abstract

**Background:**

Ambient air pollution is a serious problem in many Eastern European countries. Elevated levels of fine airborne particulate matter (PM_2.5_) pollution in the former Soviet republics relative to the rest of Europe contribute to elevated rates of disease, especially cardiovascular disease (CVD).

**Objective:**

Information on the underlying social and political causes of air pollution in Eastern Europe is important for pollution control and disease prevention.

**Methods:**

To quantify relationships between pollution, and air-pollution-related CVD, and political corruption throughout Europe and particularly in the former Soviet republics, we relied on the State of Global Air report for information on air pollution levels; on the 2019 Global Burden of Disease study (GBD) for estimates of the burden of air-pollution-related CVD; and on Transparency International (TI) for rankings of governmental corruption.

**Findings:**

Air-pollution-related CVD was responsible for an estimated 178,000 (UI: 112,000–251,000) premature deaths and for the loss of 4,010,000 (UI: 2,518,000–-5,611,000) productive years of life (DALYs) in 2019 in the former Soviet republics. A significant positive correlation (R = 0.72, p 1.7e–8) was found across Europe between air-pollution-related CVD mortality rates and national corruption rankings.

**Conclusions:**

We conclude that lack of governmental transparency, inadequate air pollution monitoring, and opposition by vested interests have hindered air pollution control and perpetuated high rates of pollution-related morbidity and mortality in the former Soviet republics. Ending corruption and modernizing industrial production will be key to air pollution and related diseases.

## Introduction

**Figure 1:**
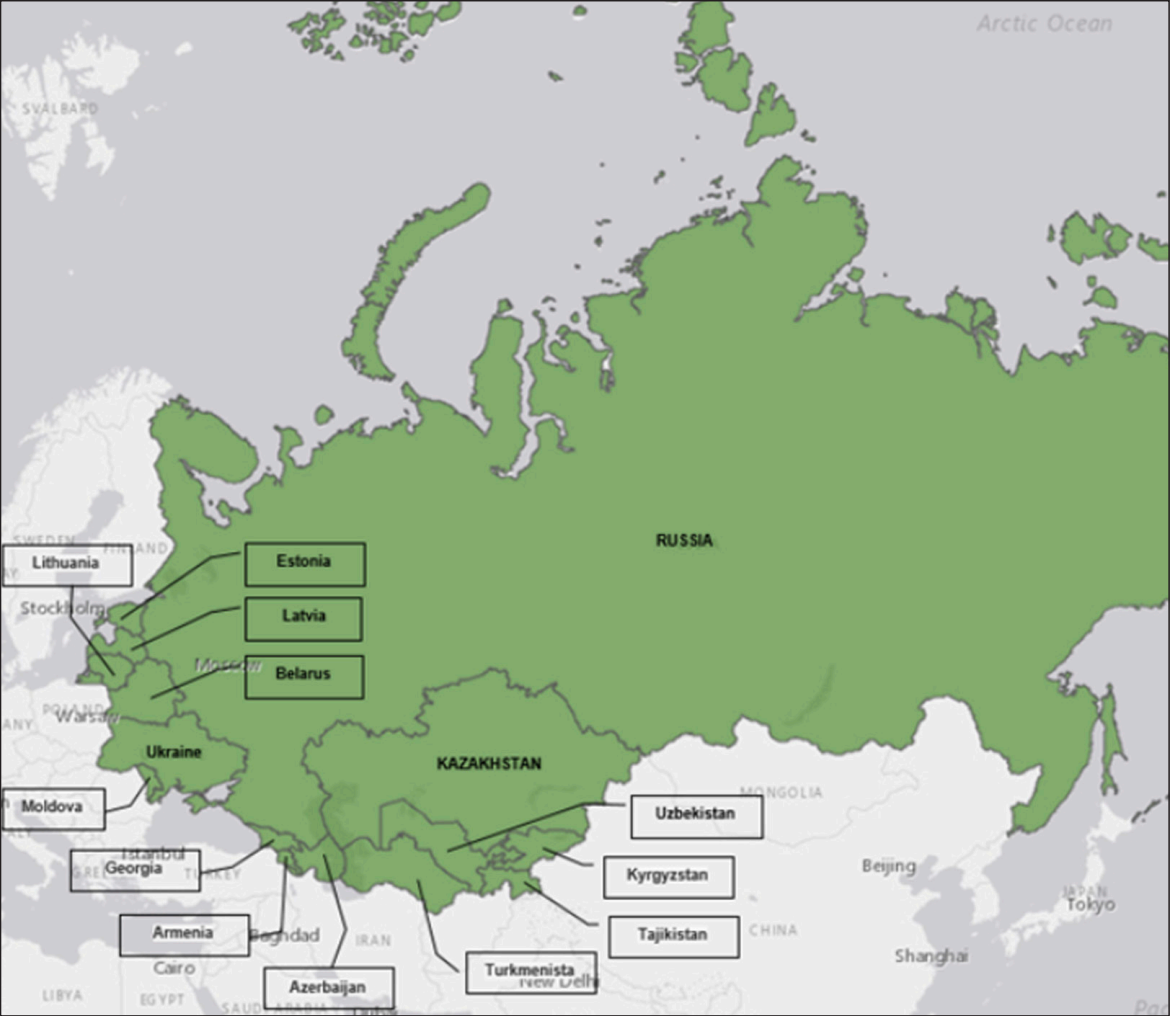
Republics formerly part of the Soviet Union.

Ambient air pollution is a leading cause of non-communicable diseases, including cardiovascular disease (CVD), chronic obstructive pulmonary disorder (COPD), stroke and cancer [1,2,3,4,5,6,7,8,9,10,11]. Ambient air pollution, including pollution by fine ambient particulate matter (PM_2.5_), is a widespread problem in the former Soviet republics of Eastern Europe (Figure 1) [12,13]. Primary sources of air pollution in Eastern Europe are an outdated, highly polluting industrial infrastructure and an ageing vehicle fleet on the region’s roads and highways [14,15].

Although air pollution levels in Eastern Europe are gradually decreasing, inadequate air monitoring and weak enforcement of air pollution standards have impeded the control of air pollution and have resulted in sharp discrepancies in air pollution levels and in rates of air-pollution-related CVD between Eastern and Western Europe [16,17].

We hypothesize that corruption and lack of governmental transparency, twin legacies of Eastern European countries’ many decades of colonization by the Union of the Soviet Socialist Republics (USSR), are associated with persistently high levels of air pollution and high rates of air-pollution-related diseases [12,18]. We hypothesize further that as Eastern European governments move past the Soviet legacy, become more democratic and less autocratic, and gain control over their legislative processes and their economies, pollution mitigation may become more effective in the future.

Control of air pollution and prevention of air-pollution-related disease in the former Soviet republics of Eastern Europe will require elucidation of the social and political factors responsible for persistently high levels of pollution. This study’s goal is to explore the root social and political causes of air pollution in Eastern Europe and suggest strategies for pollution control and prevention of pollution-related disease.

## Materials and Methods

Data on average annual population-weighted PM_2.5_ concentrations and on the proportion of the population in each country using solid fuels (surrogate measure of household air pollution) were obtained from the 2019 State of Global Air report developed by the Health Effects Institute. Estimated population-weighted ambient PM_2.5_ concentrations were derived from thousands of air pollution monitoring stations combined with satellite observations [16].

Data from the 2019 Global Burden of Disease Study (GBD) were used to estimate the burden of air-pollution-related cardiovascular disease and death in each country [13].

To assess the level of political corruption in each country, we relied on Transparency International’s (TI) Corruption Perceptions Index Ranking (CPI). This value is based on TI’s assessment of each country’s productive abilities, industrialization levels, and competitiveness in the world’s market [17]. TI conducts 13 surveys in 180 countries and gathers data measuring corruption in public sectors. TI defines “corruption as the abuse of entrusted power for private gain” and emphasizes its ability to amplify social, environmental, political, and economic injustices [19].

This study included data from all counties in the World Health Organization’s (WHO) EURO region (C) [20]. Andorra, Cyprus, Monaco, Montenegro, North Macedonia, San Marino, and Serbia were excluded from our analysis because no CPI was provided for these countries.

The software used in this analysis included Tableau and RStudio. Analysis of variance (ANOVA) analysis was used to assess deviance. Pearson’s correlation was calculated for relationships between CPI and the burden of disease and death attributable to air-pollution-related CVD.

## Results

### Air Pollution in Europe

**Figure 2:**
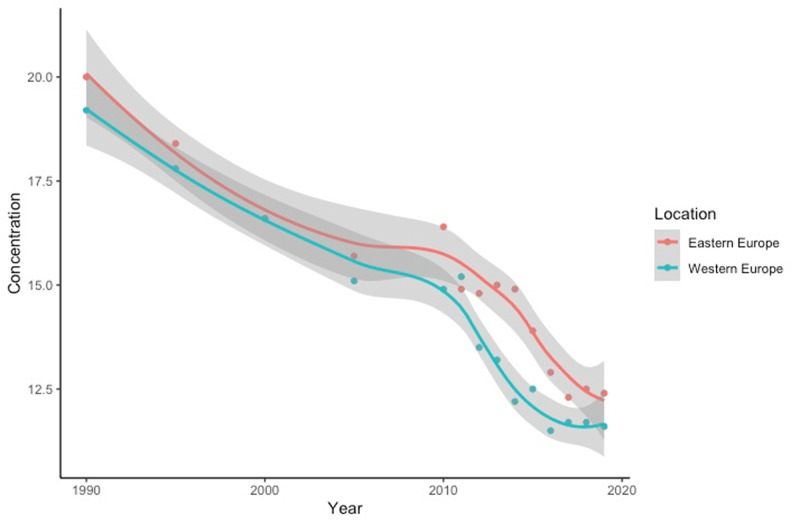
Average Annual Population-Weighted PM_2.5_ Concentration (1990–2019) [16]. Source: State of Global Air.

Air pollution levels have decreased in Eastern Europe from 20.0 µg/m^3^ in 1990 to 12.4. µg/m^3^ in 2019 (Figure 2). Particularly sharp decreases have been noted since 2010. Although pollution concentrations have declined, PM_2.5_ levels in Eastern Europe have consistently remained higher than those in Western Europe. Based on the separation of confidence bands, Western Europe’s levels of PM_2.5_ might be significantly lower than Eastern Europe’s in recent years. It is also important to note that pollution levels have been decreasing similarly in former Soviet republics, as well.

**Figure 3:**
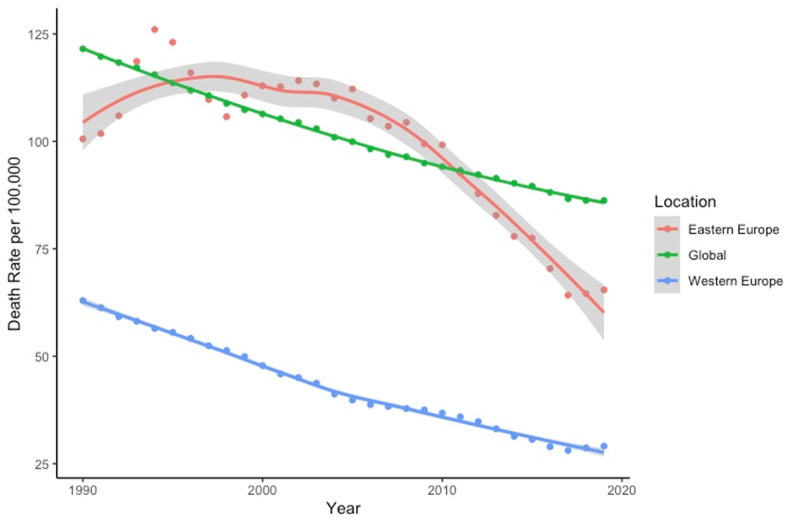
Deaths per 100,000 from all causes attributable to air pollution (1990–2019). Source: IHME.

In 1993, all-cause mortality attributable to air pollution in Eastern Europe surpassed global rates and remained elevated until it decreased sharply after 2010 (Figure 3). Rates of pollution-related disease and death in Eastern Europe have exceeded those in Western Europe throughout the past 29 years.

Table 1 shows death and DALY rates attributable to air pollution in 2019 Eastern Western Europe. Eastern Europe has dramatically higher rates under both parameters.

**Table 1:** DALY and death rates per 100,000 attributable to air pollution (2019).


COUNTRY	DEATH RATE (95% UI)	DALY RATE (95% UI)

Eastern Europe	65.44 (41.96–89.90)	1469.77 (946.28–1997.94)

Western Europe	29.09 (21.36–37.28)	568.19 (417.59–732.45)


Source: IHME.

### Cardiovascular Disease Resulting from Air Pollution

**Figure 4:**
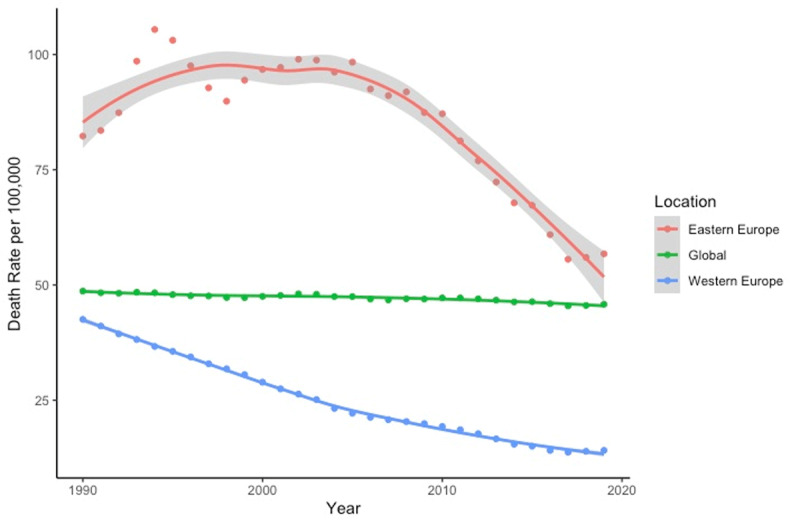
Death rates from CVD attributable to air pollution (1990–2019). Source: IHME.

Despite recent declines, Eastern European countries consistently exhibit greater mortality from air pollution related CVD compared to both Western European and global rates (Figure 4). Thus, the air-pollution-related CVD mortality rate fell from 82.29 deaths per 100,000 (UI: 41.01–125.43) in 1990 to 56.76 deaths per 100,000 (UI: 35.92–77.91) in 2019. The global death rate from air pollution related CVD decreased slightly over the same time, hovering around 46–48 deaths per 100,000. The air-pollution-related CVD mortality rate in Western Europe declined continuously from 1990 to 2019 from 42.53 (UI: 19.62–68.71) per 100,000 in 1990 to 14.13 (UI: 10.29–18.26) in 2019.

### Corruption

**Figure 5:**
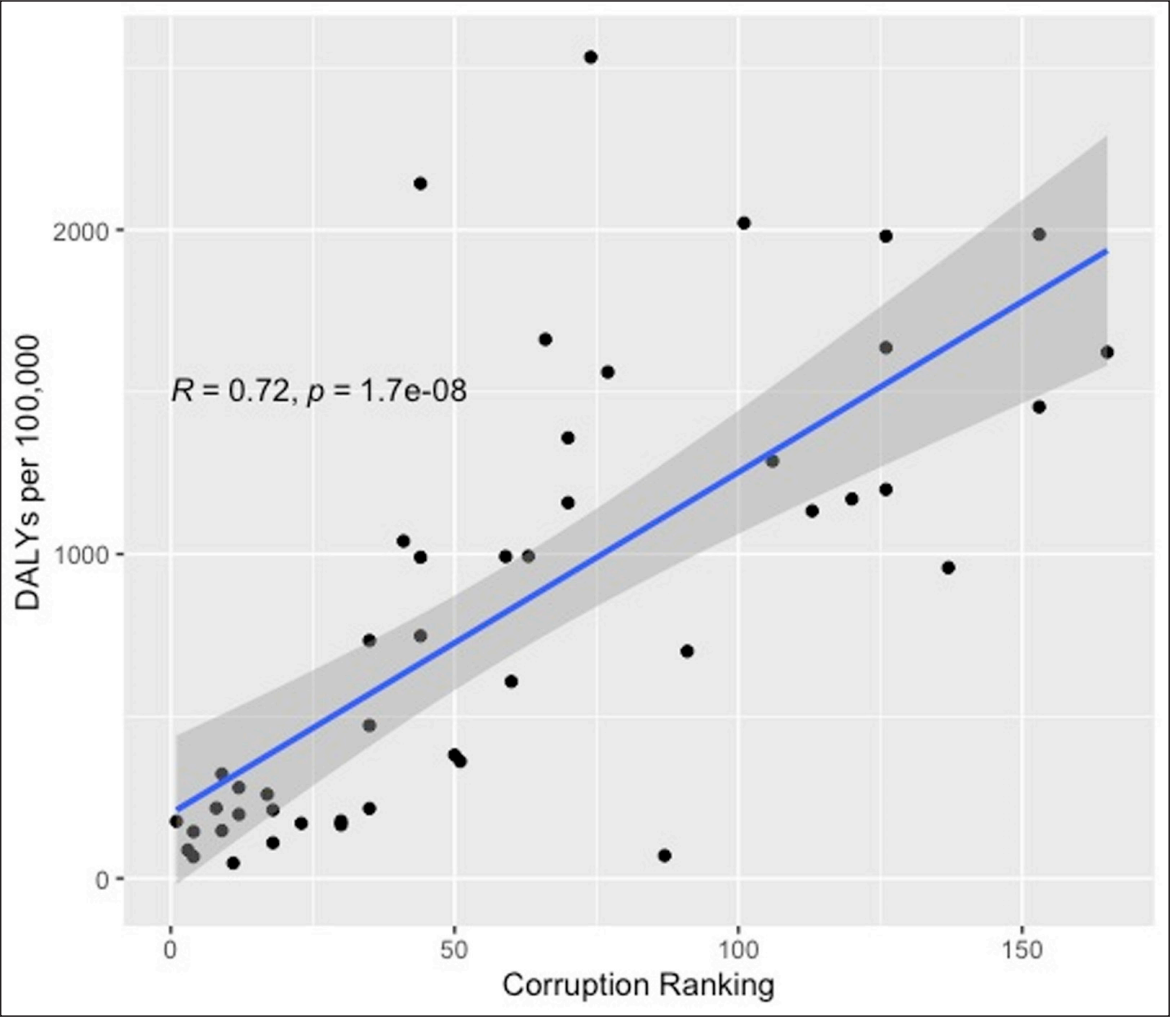
DALY rates per 100,000 from CVD attributable to air pollution by CPI.

We correlated corruption levels, as measured by CPI, with air-pollution-related CVD DALY rate per 100,000 for countries in WHO’s EURO region (Figure 5). We found a statistically significant (p = 1.7e–8) positive correlation between corruption level and pollution-related CVD across Europe with a Pearson correlation coefficient (R) of 0.72.

**Figure 6:**
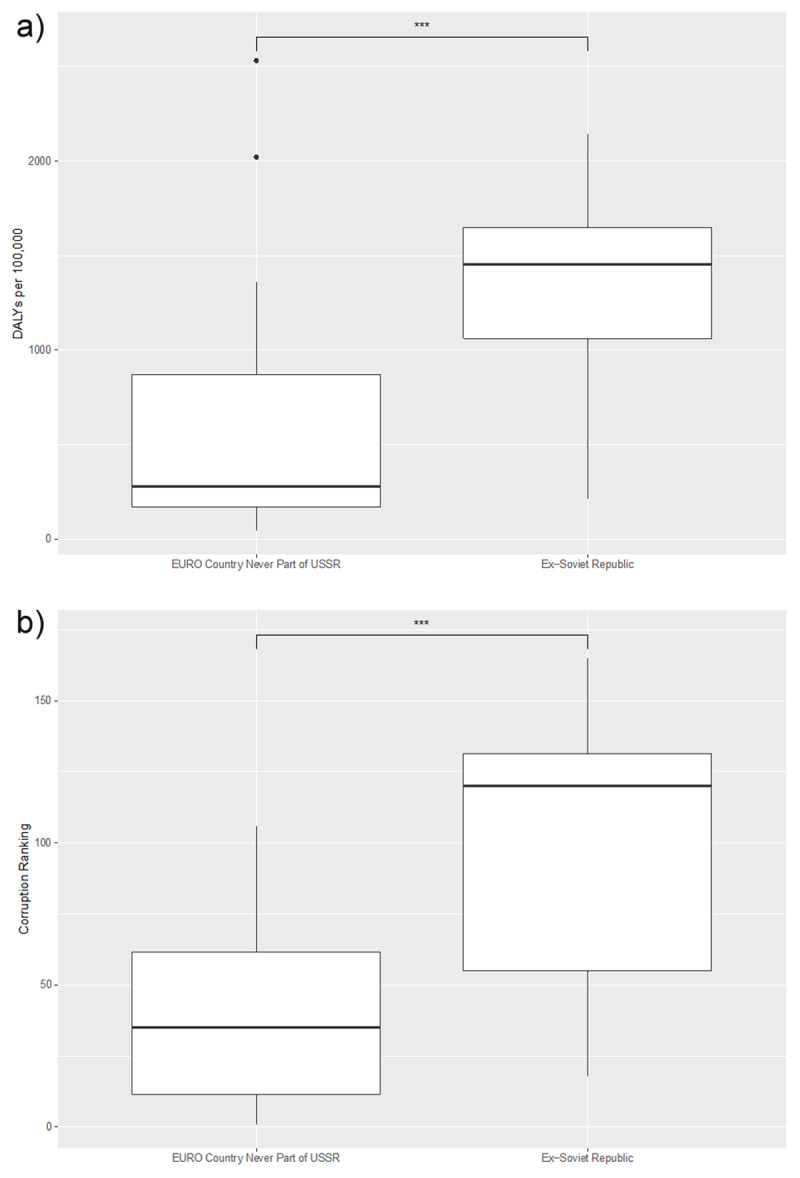
**(a)** Comparison of air-pollution-related CVD DALY rates in EURO countries never part of USSR and Ex-Soviet Republics; **(b)** Comparison of CPI in EURO countries never part of USSR and Ex-Soviet Republics. *** Indicates p-value < 0.001 from ANOVA test.

When we compared air-pollution-related morbidity with corruption between the former Soviet Republics and countries in the WHO EURO region that were never part of the USSR, we observed that both corruption and air pollution morbidity were significantly elevated in countries that were once part of the Soviet Union (Figure 6).

## Discussion

Air pollution is a significant threat to human health in Eastern Europe. For many years, this region has had a significantly higher burden of disease and premature death attributable to air pollution (Figure 3) than either Western Europe or the rest of the world. This inequity is especially visible in the former Soviet republics. Since the fall of the Soviet Union in 1991, air pollution levels within these countries have declined but have nonetheless remained higher than levels in Western Europe, as have rates of pollution-related disease and death. This is especially true in the case of CVD.

Political corruption is highly prevalent in the former Soviet republics. Ukraine, Belarus, and Georgia have particularly high levels of corruption that correlate with high levels of air pollution and air-pollution-related CVD. Since the correlation between corruption and pollution that we report in this ecological study does not prove causation, there are other factors that will need to be considered in future studies such as hypertension, tobacco use, elevated hemoglobin A1c, and dyslipidemia [21]. Nonetheless, the association is statistically very strong and merits further investigation.

The plausibility that political corruption is a powerful determinant of pollution and pollution-related disease in Eastern Europe is supported by the observation that corrupt governments can inhibit the implementation of environmental regulations, allowing for increases in pollution. Corruption within a state has been shown to decrease economic transparency and to increase the size of the hidden economy [22], economic activities that are “off the books” or illegal. It is much cheaper for industries and individuals to operate within this relatively unregulated informal sector than to comply with environmental regulations. This form of corruption is prevalent across the former Soviet Union, formerly called the Second Economy of the Soviet Union and Eastern Europe [23]. For example, vehicles that do not abide by Georgia’s attempts to mitigate air pollution are very common on roads in the country [24]. Additionally, increasing Georgian governmental regulations on businesses resulted in greater corruption in order to avoid the costs of implementing new policies [25]. Increases in corruption within governments have been shown to correlate to decreased environmental sustainability [26].

Newfound independence upon the dissolution of the Soviet Union left former Soviet republics politically and economically vulnerable. Although there have been improvements in pollution levels in the past decade, high levels of pollution continue to erode these countries’ natural environment and health today. Ending corruption and modernizing industrial production will be key to preventing air pollution and pollution-related diseases in the former Soviet republics.

## Solutions and Recommendations

### Make air pollution a top priority in Eastern Europe

The countries of Eastern Europe continue to deal with widespread political and economic crises, many of them the consequence of continuing tensions with Russia [27]. These tensions have been brought into sharp relief by Russia’s recent unjustified declaration or war on Ukraine. They limit nations’ abilities to grapple with issues surrounding air pollution. However, if these nations are to grow their economies, increase population health and overcome the legacy of Soviet domination, they will need to make air pollution control and health protection top priorities.

### Promote clean renewable energy

Nations around the world have been moving away from fossil fuels toward renewable energy to combat climate change and reduce pollution. Renewable technologies improve air quality and benefit health, and they also improve the economy by creating more jobs and establishing stability and reliability [28,29,30]. A further incentive for nations to transition from fossil fuels to renewable energy is that the cost of generating electricity from solar power has declined by 90% in the past decade and in the same time the cost of generating electricity from wind power has fallen by 70% [30].

Strategies for reducing road traffic and traffic-related air pollution are a second key strategy for combatting pollution. These include raising parking fees, mandated vehicle inspections, fuel taxes, redesigning cities to improve public transit and creation of paths for cycling and walking.

### Promote and enforce current air pollution control policies

In countries that suffer from high levels of air pollution and pollution-related disease, it is necessary to properly enforce the appropriate legislation and standards to control air pollution. Unenforced standards do not protect health. Inadequate training and corruption impede the effectiveness of enforcement [31].

### Increase transparency of governments and economies

Countries with large “hidden” economies and corrupt governments do not generally publicize their internal affairs. This lack of transparency keeps the public from knowing about pollution levels and pollution control policies. In-creased transparency is critically important for overcoming these barriers to knowledge.

Specific steps that nations can take to increase transparency regarding pollution include establishing and publicizing up-to-date and adequate air quality metrics and providing real-time information on pollution levels to the public. Such information empowers citizens and is a powerful tool for improving health and reducing inequality [22].

### Promote clean industrialization

Without proper industrialization policies and enforcement of air quality standards, the benefits of economic growth can be undercut by increases in harmful air pollutants and the resulting pollution-associated economic losses. The United Nations Environment Programme (UNEP) has published guidelines for Inclusive and Sustainable Industrial Development, highlighting strategies to properly industrialize while achieving long-term health and ecologic benefit and environmental sustainability. In this context, pollution prevention is seen as a source of economic competitiveness – an investment rather than an unwanted expense [32].

### Publish annual local and national air pollution evaluations and reports

The former Soviet republics of eastern Europe highlighted in this report have all published air quality reports since 2015 with the support of UNEP. However, many of these national reports are incomplete, and key data points are not included [33,34,35]. The countries that published more complete and comprehensive air quality reports experienced lower rates of pollution-related morbidity and mortality – further evidence of the link between governmental transparency and clean air.

To improve air quality reporting in countries where reporting is currently in-complete, more air quality monitoring stations need to be placed and findings from these pollution monitors need to be frequently reviewed and communicated in real time and without censorship to the public.

### Educate health professionals and the public about the hazards of air pollution and its links to CVD

For air pollution to improve, people need to be educated about the hazards of air pollution and about how their actions can help reduce pollution. Governments can implement initiatives that educate people about air pollution and how it can damage their health. This information can motivate people to change habits that will directly improve air quality as well as galvanize them to engage with governments (local and national) and advocate for change [36].

### Incentivize limiting emission sources and moving to renewable energy

Countries such as Norway and France provide monetary compensation, select tax exemptions, and the virtual elimination of fuel costs for people with electric vehicles. This creates incentives for people to own electric vehicles instead of petrol and other fuel powered cars. These actions reduce traffic-related air pollution and improve health [37]. They provide a road map for the countries of Eastern Europe.

## Conclusions

The development and enforcement of air pollution control laws and policies is a critically important strategy for decreasing the burden of disease and death due air pollution. In the United States, laws and policies implemented since passage of the Clean Air Act in 1970 have reduced air pollution levels by 70% and brought about a corresponding reduction in rates of pollution-related disease. Enormous economic benefits have also accrued with an estimated benefit of $30 (USD) for every $1 invested in pollution control [38].

In the former Soviet republics, implementation of the pollution control measures we suggest here must also accompany a transition to less corrupt and more transparent governments if lasting control of pollution is to be achieved. Reductions in corruption and increases in justice and governmental transparency correspond to reduced deaths caused by air pollution and bring about striking reductions in the association of morbidity and mortality due to air-pollution-related CVD.

## Data accessibility statement

Data for CVD and air pollution related disease and death was gathered from the 2019 GBD database using the GBD Results Tool. This can be found at http://ghdx.healthdata.org/gbd-results-tool. The PM_2.5_ concentration data was used from the 2019 State of Global Air Report found at https://www.stateofglobalair.org/sites/default/files/soga_2019_report.pdf. Corruption Rankings were accessed through the Transparency International database at https://www.transparency.org/cpi/2020. The list of countries in the EURO region was provided by the World Health Organization at https://www.euro.who.int/en/countries.
